# Negative-mass exciton polaritons induced by dissipative light-matter coupling in an atomically thin semiconductor

**DOI:** 10.1038/s41467-023-36618-6

**Published:** 2023-02-23

**Authors:** M. Wurdack, T. Yun, M. Katzer, A. G. Truscott, A. Knorr, M. Selig, E. A. Ostrovskaya, E. Estrecho

**Affiliations:** 1 ARC Centre of Excellence in Future Low-Energy Electronics Technologies and Department of Quantum Science and Technology, Research School of Physics, The Australian National University, Canberra, ACT 2601 Australia; 2 Department of Materials Science and Engineering, Monash University, Clayton, Victoria 3800 Australia; 3 Songshan Lake Materials Laboratory, Dongguan, 523808 Guangdong China; 4 Institute of Physics, Chinese Academy of Science, Beijing, 100190 China; 5 Nichtlineare Optik und Quantenelektronik, Institut für Theoretische Physik, Technische Universität Berlin, 10623 Berlin, Germany; 6 Department of Quantum Science and Technology, Research School of Physics, The Australian National University, Canberra, ACT 2601 Australia

**Keywords:** Two-dimensional materials, Polaritons

## Abstract

Dispersion engineering is a powerful and versatile tool that can vary the speed of light signals and induce negative-mass effects in the dynamics of particles and quasiparticles. Here, we show that dissipative coupling between bound electron-hole pairs (excitons) and photons in an optical microcavity can lead to the formation of exciton polaritons with an inverted dispersion of the lower polariton branch and hence, a negative mass. We perform direct measurements of the anomalous dispersion in atomically thin (monolayer) WS_2_ crystals embedded in planar microcavities and demonstrate that the propagation direction of the negative-mass polaritons is opposite to their momentum. Our study introduces the concept of non-Hermitian dispersion engineering for exciton polaritons and opens a pathway for realising new phases of quantum matter in a solid state.

## Abstract (web summary)

Dissipation and losses are normally perceived as detrimental to the performance of electronic and photonic devices. Here, the authors demonstrate dissipative coupling between excitons and photons in optical microcavities which they then use to create polaritons with a negative effective mass.

## Introduction

Losses are ubiquitous in nature and are usually perceived as detrimental to the performance of electronic and photonic devices. However, recent understanding of the physics of non-Hermitian systems with loss and gain has led to the possibility of novel properties and functionalities by judicious control of losses. This concept is most powerfully demonstrated in non-Hermitian photonics^1–3^, where non-Hermitian spectral degeneracies (exceptional points) and associated symmetry-breaking transitions fundamentally change the laws of wave propagation and scattering. Although the study of non-Hermitian physics in quantum electronic systems remains difficult, significant progress has been achieved in hybrid photonic systems, where photons are strongly coupled to electronic excitations in a solid state to form exciton polaritons, part-light part-matter hybrid quasiparticles^4^. Nontrivial topology of the eigenstates^5,6^ and mode selectivity^7^ in the vicinity of the exceptional points, band engineering^8^ and nonlinear localisation^9^ in non-Hermitian lattices, emergence of non-Hermitian topology^10^ and divergent quantum geometric metric near an exceptional point^11^ have been demonstrated in the strong light-matter coupling regime.

Here, we dramatically modify the exciton–polariton dispersion by exploiting a previously undetected non-Hermitian component of exciton–photon interaction called dissipative coupling^12^. Our microscopic theory shows that this type of coupling can arise from the interplay of exciton–phonon scattering in monolayers of transition metal dichalcogenide crystals (TMDCs) and photon losses. Also known as external coupling via the continuum^13^, dissipative coupling leads to level attraction or clustering^13,14^ and resonance trapping^15^ in other physical systems. This is in contrast to the well-known coherent (or internal) coupling which always leads to level repulsion. We show theoretically that the interplay between the coherent and dissipative light-matter coupling results in an inverted dispersion of exciton polaritons in a planar microcavity with an embedded monolayer TMDC. We directly measure this anomalous dispersion in several planar microcavities with integrated monolayer WS_2_^16^ at room temperature, and demonstrate the negative-mass transport of exciton polaritons. The key role of the exciton–phonon scattering in the dissipative coupling mechanism is further confirmed by temperature-dependent measurements.

## Results

### Theory

To demonstrate the principle of non-Hermitian dispersion engineering, we start with an effective Hamiltonian given by *H* = *H*_0_ − *iWW*^†^^15^ describing the coherent (internal) and dissipative (external) coupling of cavity photons $\left|C\right\rangle$ and excitons $\left|X\right\rangle$:

1 $$H=\left(\begin{array}{cc}{E}_{c}&V\\ V&{E}_{x}\end{array}\right)-i\left(\begin{array}{cc}\sqrt{{\gamma }_{c}}&\sqrt{{g}_{x}}\\ \sqrt{{g}_{c}}&\sqrt{{\gamma }_{x}}\end{array}\right)\left(\begin{array}{cc}\sqrt{{\gamma }_{c}}&\sqrt{{g}_{c}}\\ \sqrt{{g}_{x}}&\sqrt{{\gamma }_{x}}\end{array}\right).$$

Here, the Hermitian term *H*_0_ models the coherent coupling of excitons and photons with the bare energies *E*_*c*,*x*_, respectively. The coherent coupling strength *V* is proportional to the exciton oscillator strength and the overlap of the exciton dipole with the confined electric field of the cavity photon^4^. The matrix *W* describes the external coupling to two dissipative channels in the system^15^: *γ*_*c*_ is the coupling of cavity photons to the continuum of states outside the cavity due to the imperfect mirrors limiting their lifetimes, *γ*_*x*_ is the coupling to radiative and non-radiative channels resulting in the homogeneous linewidth broadening of excitons^17,18^, and *g*_*x*,*c*_ quantifies the effective dissipative coupling between excitons and cavity photons via the two decay channels.

This non-Hermitian, phenomenological Hamiltonian, Eq. (1), is motivated by a full microscopic model (see Supplementary Note 1). In a self-consistent theory without free parameters, with all values from ab initio calculations, we show that the off-diagonal dissipation terms in *W* (cp. Eq. (1)) arise due to the mixing of the two decay channels: (i) the dissipation of energy from the excitons via phonon scattering events in the TMDC, i.e. due to the phonon bath, leading to *g*_*x*_ and (ii) the energy loss due to leakage of photons out of the cavity leading to *g*_*c*_. Only for nonzero off-diagonal dissipation terms in Eq. (1) one can observe level attraction and anomalous dispersion as described below.

Remarkably, when the coupling to the phonon bath is ‘turned off’ in the microscopic model, the effects arising from the dissipative coupling disappear (see Supplementary Note 1). Since the exciton–phonon scattering is known to be significant in TMDCs^17–20^, we expect strong effects of dissipative coupling via this channel in this material system. Furthermore, for all-dielectric TMDC microcavities at room temperature, we expect *g*_*c*_ to be much smaller than *g*_*x*_, since photon losses (affecting the cavity photon linewidth) are much weaker than phonon-induced losses (affecting the exciton linewidth)^16,21^. Hence, herein, we set *g*_*c*_ = 0 and *g*_*x*_ = *g*.

The exciton–photon coupling in Eq. (1) gives rise to the complex upper (*U*) and lower (*L*) eigenvalue branches:

2 $${E}_{U,L}-i{\gamma }_{U,L}=\langle \tilde{E}\rangle \pm \frac{1}{2}\sqrt{{\left(\Delta -i\delta \right)}^{2}+4{\left(V-i\sqrt{g{\gamma }_{x}}\right)}^{2}},$$

where $\langle \tilde{E}\rangle=\langle E\rangle -i\langle \gamma \rangle$ is the mean complex eigenvalue with 〈*γ*〉 = (*γ*_*c*_ + *g* + *γ*_*x*_)/2, Δ = *E*_*c*_ − *E*_*x*_ is the bare energy difference and *δ* = *γ*_*c*_ + *g* − *γ*_*x*_. The eigenvalues and eigenvectors will simultaneously coalesce at the exceptional point when *V* = ∣*δ*∣/2 and $\Delta=2\sqrt{g{\gamma }_{x}}$. Note that when *g* = 0, the non-Hermitian term −*iWW*^†^ simply describes the decay rates *γ*_*c*,*x*_/ℏ of the bare (uncoupled) cavity photon and exciton, respectively.

**Fig. 1:**
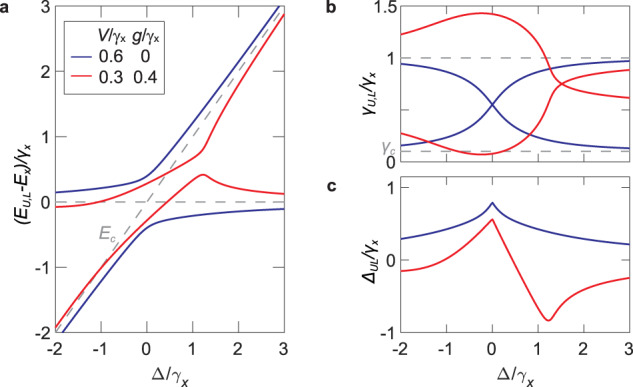
Coherent and dissipative coupling. **a** Energy *E*_*U*,*L*_ and **b** linewidth *γ*_*U*,*L*_ dynamics for different values of coherent *V* and dissipative *g* coupling strengths. Dashed lines in **a** are the bare exciton *E*_*x*_ and cavity *E*_*c*_ energies while those in (**b**) correspond to the bare exciton *γ*_*x*_ and cavity *γ*_*c*_ decay rates. **c** Energy shift Δ_*UL*_ (see text) for the parameters used in (**a**) and (**b**). In all cases, we tuned the cavity energy *E*_*c*_ while fixing *E*_*x*_ and *γ*_*c*_ = 0.1*γ*_*x*_.

The level and linewidth dynamics for different strengths of *V* and *g* are shown in Fig. 1a, b. The general behaviour in Δ-*g* parameter space is discussed in Supplementary Note 2. We focus on the strong coupling regime at *V* > ∣*δ*∣/2 characterised by energy anticrossings and linewidth crossings. In this regime, the eigenstates correspond to the hybrid exciton–polariton quasiparticles^4,22–24^. The corresponding shifts of the polariton energies from the bare exciton and photon energies, defined as ${\Delta }_{UL}={\mathfrak{Re}}({E}_{U}-{E}_{L})-\Delta$, is plotted in Fig. 1c as a function of Δ. In the purely coherent coupling regime (*g* = 0), the energies exhibit level repulsion (positive Δ_*UL*_) with a maximum value at resonance (Δ = 0) where the linewidths cross. The level repulsion decreases monotonically with ∣Δ∣, reminiscent of the familiar Hermitian limit, *γ*_*c*,*x*_, *g* → 0^4^.

The behaviour of the energy levels and linewidths is drastically modified when the dissipative coupling *g* is introduced. The linewidth crossing shifts towards higher Δ leaving a linewidth repulsion close to resonance, which leads to the so-called resonance trapping of the long-lived state^15^. Remarkably, the energy shift Δ_*UL*_ becomes negative for a wide range of Δ, indicating level attraction which peaks at a positive $\Delta \approx 2\sqrt{g{\gamma }_{x}}$ before monotonically decreasing. This strongly Δ-dependent energy shift is responsible for the anomalous dispersion of exciton polaritons presented in this work.

**Fig. 2:**
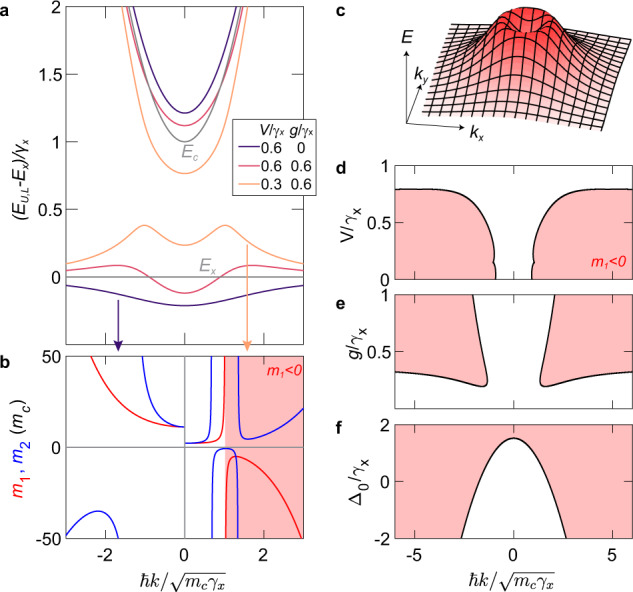
Anomalous dispersion of exciton polaritons. **a** Dispersion for different values of coherent and dissipative coupling strenghs at fixed exciton–photon detuning Δ_0_ = *γ*_*x*_. Thin grey lines are the bare exciton *E*_*x*_ and cavity photon *E*_*c*_ dispersions. **b** *k*-dependent mass parameters *m*_1_ (red) and *m*_2_ (blue) corresponding to the two dispersions in (**a**) (see arrows). *m*_1_ < 0 in the shaded region. **c** Lower polariton dispersion in two-dimensional *k*-space corresponding to (**a**) with *V* = 0.3*γ*_*x*_ and *g* = 0.6*γ*_*x*_. **d**–**f** Negative-mass (*m*_1_ < 0) regions (shaded) in *k*-space as a function of **d** *V* for *g* = 0.6*γ*_*x*_, Δ_0_ = *γ*_*x*_, **e** *g* for *V* = 0.6*γ*_*x*_, Δ_0_ = *γ*_*x*_, and **f** Δ_0_ for *V* = 0.3*γ*_*x*_, *g* = 0.6*γ*_*x*_.

To describe the exciton–photon dispersion, we approximate the cavity photon dispersion as^4^ *E*_*c*_(*k*) = *E*_0_ + ℏ^2^*k*^2^/2*m*_*c*_, where *E*_0_ is the cavity resonance energy at normal incidence, ℏ*k* is the momentum along the plane of the cavity, and *m*_*c*_ is the in-plane effective mass of the cavity photon. The exciton energy *E*_*x*_ is approximately constant within the relevant momentum range probed here. Typical dispersion curves at a positive exciton–photon detuning, Δ_0_ = *E*_0_ − *E*_*x*_, are presented in Fig. 2a. Without the dissipative coupling (*g* = 0), the dispersion features repelling branches corresponding to the well-known upper and lower exciton polaritons^4^, where the lower branch is always redshifted from the exciton line. With increasing dissipative coupling strength *g*, level attraction starts to dominate, firstly starting at higher *k*. When *g* is large enough compared to *V*, the entire lower polariton branch is blueshifted from the exciton line.

Note that, for this set of parameters, the strongest level attraction occurs at a finite *k*, i.e., where $\Delta (k) \, \approx \, 2\sqrt{g{\gamma }_{x}}$ and the level attraction peaks (see Fig. 1c). However, for a very large exciton–photon detuning, i.e. ${\Delta }_{0} \, > \, 2\sqrt{g{\gamma }_{x}}$, the strength of level attraction monotonically decreases for all *k*, resulting in a single inversion peak at *k* = 0 (see Supplementary Note 3).

To characterise the dispersion, we define the mass parameters^25^ ${m}_{1}(k)={\hslash }^{2}k{[{\partial }_{k}E(k)]}^{-1}$, which determines the group velocity *v*_*g*_ = ℏ*k*/*m*_1_, and ${m}_{2}(k)={\hslash }^{2}{[{\partial }_{k}^{2}E(k)]}^{-1}$, which determines the acceleration due to an external field. Note that *m*_1_ is only negative around the inverted dispersion whereas *m*_2_ switches signs at the inflection points. The masses are plotted in Fig. 2b for the lower branches in Fig. 2a (indicated by arrows) with and without dissipative coupling. When *g* = 0, *m*_1_ is positive for all momenta and *m*_2_ is only negative at finite *k*, a known feature of exciton polaritons^25^. This is in stark contrast to the case with *g* ≠ 0, where both masses become negative near the inversion peak. While *m*_2_ switches back to positive sign, *m*_1_ remains negative for the plotted range of momenta, shown by the shaded region in Fig. 2b. Note that the sign of the *m*_1_ and *m*_2_ for the upper branch is largely unaffected by *g*.

It is important to point out that the inverted dispersion is isotropic, forming a ring in *k*-space, as shown in Fig. 2c. This is distinct from the inverted bands in periodic band structures, where the inversion peaks are localised at high-symmetry points only^26^.

We further analyse the Δ_0_-*V*-*g* parameter space as a function of ℏ*k* to determine under which conditions the inverted dispersion appears. Notably, the model predicts that the negative-mass regions disappear when coherent coupling significantly dominates over dissipative coupling, as shown by the plots in Fig. 2d, e. Hence, either *V* has to be decreased or *g* increased to observe the inverted dispersion. The negative-mass regime also persists for a wide range of the exciton–photon detuning Δ_0_, as shown in Fig. 2f. However, the momentum range corresponding to the negative mass increases with the detuning Δ_0_ and the dispersion becomes completely inverted at large positive Δ_0_/*γ*_*x*_. In summary, positive exciton–photon detunings Δ_0_ and strong *g* with respect to *V* favour the negative-mass regime in our system.

### Anomalous dispersion experiments

To demonstrate the effects of the anomalous dispersion experimentally, we fabricated several planar microcavities with integrated monolayer WS_2_ at positive exciton–photon detunings and with reduced coherent coupling strength *V*. We achieved this by using substrate engineering and our recently developed technology for integrating monolayer WS_2_ into polymethyl-methacrylate (PMMA)/SiO_*x*_ spaced planar microcavitites^16^. The relative exciton oscillator strength, and hence *V*, of the monolayer is weakened after the transfer onto our distributed Bragg reflector (DBR) and further material deposition (see Supplementary Note 4).

**Fig. 3:**
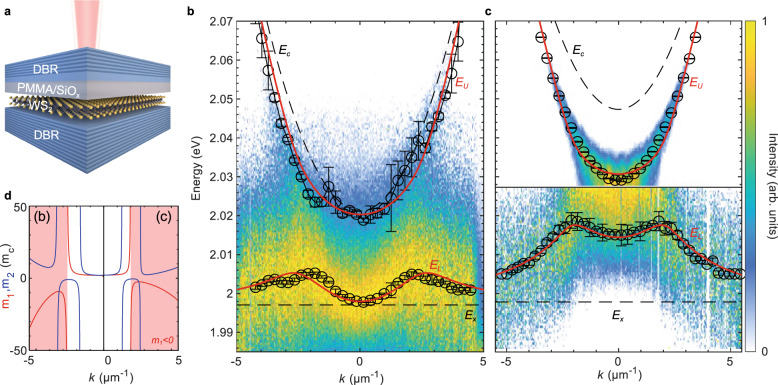
Experimental realisation of negative mass. **a** Schematics of the fabricated microcavity with embedded monolayer WS_2_. **b**, **c** Momentum-resolved PL spectra of two samples with different values of the exciton–photon detuning, Δ_0_. The maximum intensity at each *k* value is scaled to unity to visualise the shape of the lower branch. In panel **c**, the normalisation of the spectrum is performed for *E* < 2.028 eV to suppress the strong emission from the upper branch. The black circles are the fitted peak positions of the two branches. The solid red lines are the fitted dispersion of the upper (*E*_*U*_) and lower (*E*_*L*_) polaritons and the black dashed lines are the bare microcavity photons (*E*_*c*_) and excitons (*E*_*x*_). Error bars in **b**, **c** represent 95% confidence interval. **d** Mass parameters (red) *m*_1_ and (blue) *m*_2_ of the lower branches in panel (**b**) and panel (**c**), with the negative-mass regions shaded in red.

Figure 3a illustrates the sample design. Here, the WS_2_ monolayer is mechanically transferred onto the bottom DBR protected with PMMA against further deposition of the thin SiO_*x*_ spacer and the top DBR (see ‘Methods’). The thin SiO_*x*_ spacer allows us to carefully adjust the exciton–photon detuning Δ_0_ and the two distributed Bragg reflectors enable a Q-factor of above 10^3^ (see Supplementary Note 4).

The momentum-resolved photoluminescence (PL) spectra of the room temperature polariton emission in two samples with different Δ_0_ are shown in Fig. 3b, c, together with the extracted peak energies of the lower and upper exciton–polariton branches. The observed PL intensities are dictated by the photonic Hopfield coefficients and thermalisation of polaritons^27^. Hence, the PL of the upper branch in Fig. 3c, which is highly photonic at such a large positive Δ_0_, strongly dominates the emission of the structure and decreases at larger energies due to thermalisation. To highlight the emission of the lower polariton branch in Fig. 3b, c we scaled the maximum values of the PL spectra to unity at each value of *k*. In Fig. 3c we defined a cut-off energy for normalisation at *E* ≈ 2.028 eV due to the strongly dominating emission from the upper branch. As seen in Fig. 3b, c, the lower branch energy decreases towards *E*_*x*_ at large momenta, demonstrating the level attraction shown in Fig. 2a. We also observe similar behaviour in reflectance measurements (see Supplementary Note 6 for more details).

The complex-valued dispersion branches are extracted by fitting the spectrum at each value of *k* using a two-peak Voigt function, with the peak energy and linewidth corresponding to the real and imaginary parts of the complex eigenvalues of the system Hamiltonian, respectively (see Supplementary Note 5). The *k*-dependence of the extracted peaks were then fitted using the model Eq. (2), with the fitting results presented as red solid lines in Fig. 3b, c. Here, the energies and linewidths of the cavity photons were extracted from the reflectivity measurements of the empty microcavities next to the monolayers (see Supplementary Note 4), and fixed for the fits, with the energies shifted by approximately −8 meV due to the optical thickness of the monolayer. The exciton linewidths and energies are expected to change after the deposition of the top DBR^16^, thus, these values were chosen as free-fitting parameters together with the coherent and dissipative coupling strengths *V* and *g*. For Fig. 3b, the fitting yields the values: Δ_0_ = (24 ± 2) meV, *γ*_*x*_ = (31 ± 12) meV, *V* = (13 ± 6) meV and *g* = (18 ± 9) meV, and for Fig. 3c, Δ_0_ = (51 ± 1) meV, *γ*_*x*_ = (42 ± 8) meV, *V* = (12 ± 5) meV and *g* = (23 ± 4) meV.

The values of the parameters for the two samples are consistent (within errors) and satisfy the condition 2*V* > ∣*δ*∣, confirming that the samples operate in the strong coupling regime and thus host exciton polaritons. However, due to the suppressed exciton oscillator strength in our samples^16^ the coupling strength *V* is smaller than *g*, leading to level attraction and the anomalous dispersion. The level attraction at *k* = 0 is stronger in the more positively detuned sample. This agrees with the calculations shown in Fig. 1c, where the magnitude of level attraction increases as $\Delta /(2\sqrt{g{\gamma }_{x}})$ approaches unity. Indeed, ${\Delta }_{0}/(2\sqrt{g{\gamma }_{x}})$ is ~0.8 (~0.5) for the more (less) positively detuned sample. Since ${\Delta }_{0}/(2\sqrt{g{\gamma }_{x}}) \, < \, 1$ in these samples, the maximum level attraction occurs at a larger Δ and *k*, and therefore, the inversion peaks in these samples are located at a finite momentum, endowing a negative mass *m*_1_ to the lower polaritons with momenta *k* ≳ ± 2.7 μm^−1^ for Fig. 3b and *k* ≳ ± 2 μm^−1^ for Fig. 3c.

Note that in samples with the same detuning, ${\Delta }_{0}/(2\sqrt{g{\gamma }_{x}})$ can be made larger than 1 by decreasing dissipative coupling *g* or narrowing the exciton linewidth *γ*_*x*_, which results in suppressed level attraction and an inversion peak located *k* = 0 (see Fig. 2f). We demonstrated this effect experimentally in a sample made using a high-quality DBR and a microcavity fabrication technique that does not reduce the oscillator strength of the monolayer exciton and therefore preserves the coherent coupling *V*^21,28^ (see Supplementary Note 7). In this sample, which has the same exciton–photon detuning as that shown in Fig. 3c, we observe much less attraction compared to the samples with weakened *V* discussed above. This agrees with our model, where the effect of dissipative coupling on the dispersion, i.e., level attraction, depends on the strength of coherent coupling. Nevertheless, the observed level attraction, albeit weak, suggests that dissipative exciton–photon coupling is ubiquitous in monolayer TMDCs but is often screened by the strong coherent exciton–photon coupling.

### Negative-mass dynamics

**Fig. 4:**
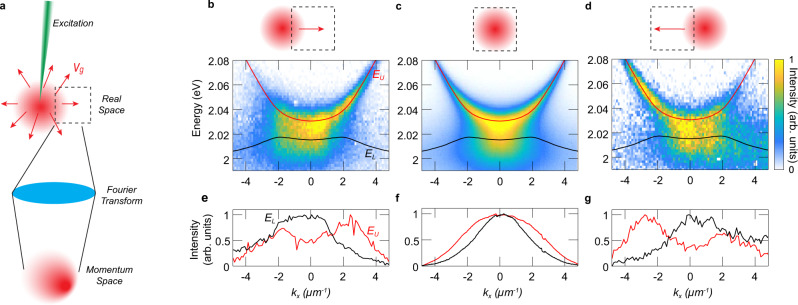
Negative mass effect on polariton dynamics. **a** Schematic of the creation of polaritons in real space (red) by a tight pump laser (green). The polaritons move away from the pump spot with a certain group velocity **v**_*g*_ (red arrows). A real space filter (dashed box) is used to isolate the detection area in real space before Fourier-transforming to measure the momentum space distribution. **b**–**d** Experimental angle-resolved spectra along *k*_*x*_ for different configurations. The fitted upper (*E*_*U*_) and lower (*E*_*L*_) polariton branches extracted from Fig. 3b are shown on top of the dispersion measurements for guidance. Insets show the position of the filter displaced along the *x*-direction with respect to the pump spot (arrows represent the average **v**_*g*_ inside the detection window). **e**–**g** Momentum distributions of the (black) lower and the (red) upper branch.

Finally, we show that the anomalous dispersion has a dramatic effect on the dynamics of the lower polaritons using the more positively detuned sample from Fig. 3c. In contrast to positive mass particles, such as the upper polaritons, negative-mass particles move in the opposite direction to their momentum ℏ**k**, i.e. their group velocity is opposite to their momentum, as given by the relation *m*_1_**v**_*g*_ = ℏ**k**. To demonstrate this behaviour in the experiment, we excite the sample with a tightly-focused off-resonant laser spot, which results in a spatially localised distribution of polaritons with a wide range of momenta, as shown by the distributions in Fig. 3b. Polaritons will then move away from the excitation spot in the direction determined by their group velocity **v**_*g*_ (see Fig. 4a). Hence, polaritons displaced to the positive (negative) *x* direction with respect to the spot must have an average velocity towards the same direction^21^. We then measure the momentum distribution of the polaritons displaced from the laser spot by Fourier transforming only a small region in real space (see Fig. 4b–g) using a spatial filter (see ‘Methods’).

Polaritons directly at the excitation spot, measured with a filter centred on the spot (see inset of Fig. 4c), feature a symmetric momentum distribution for both upper and lower branches, as shown by the momentum-resolved PL spectrum in Fig. 4c. This is clearly seen in Fig. 4f, where we directly plot the momentum distribution of the two branches by separating the spectrum at ~2.02 eV. To obtain these plots, we integrate the intensities for each panel above and below the cut-off energy, and scale the respective maximum values to unity. The PL spectrum shows a dramatic change in the distribution for particles displaced to the right of the spot, as shown in Fig. 4b, e. The upper polariton emission is skewed to the right, i.e., the average momentum has the same direction as its group velocity, but remarkably, the lower polariton emission is skewed to the left, i.e., its average momentum is opposite to the particle displacement or the group velocity. The additional peak to the left of the upper polariton distribution likely arises from the tail of the lower polariton distribution, which has a much larger linewidth. This opposite behaviour of momentum and group velocity of the lower polaritons is consistently observed to the left of the laser spot, as demonstrated by Fig. 4d, g, where the particles displaced to the left, or with leftward group velocity, have the opposite, rightward average momentum. In all configurations, the upper polariton branch with the positive mass behaves as expected, i.e., the group velocity and momentum are parallel. Therefore, we have confirmed the negative-mass dynamics of the polaritons using a mixture of particles with distinct signs of effective masses *m*_1_.

## Discussion

In summary, we have observed dissipative coupling of excitons and photons in monolayer TMDCs (WS_2_) embedded in planar microcavities at room temperature. This coupling drastically modifies the dispersion of the coupled exciton–photon system, leading to an anomalous (inverted) dispersion for the lower polariton branch. The anomalous dispersion arises when the dissipative coupling between excitons and cavity photons overcomes their coherent coupling, which leads to a negative-mass regime in a large range of momenta at a positive exciton–photon detuning. We have demonstrated the negative-mass effect on the dynamics of polaritons, resulting in the opposing directions of group velocity and momentum. This dynamics should also occur for trion polaritons with anomalous dispersion^29,30^.

The dissipative coupling causing the observed effects can arise from the coupling of both excitons and photons to the same decay channel^15^, or to a third dissipative mode^31^. As discussed above and confirmed by our microscopic theory (see Supplementary Note 1), for TMDCs, such as WS_2_, this coupling likely arises from exciton–phonon interactions^18,19,32^. Recent studies have already shown that phonons play a significant role in exciton–photon interactions^20^. Furthermore, our hypothesis is supported by the temperature-dependent measurements shown in Supplementary Note 6, where the polariton dispersion exhibits a transition from level attraction to level repulsion by lowering the temperature. This is likely because the exciton–phonon interactions and the relative values of dissipative coupling strength *g* versus the coherent coupling strength *V* diminish at lower temperature. Dissipative coupling can also arise via coupling to a ‘hidden’ photon mode^31^, but this mode with opposite polarisation is not present in our planar DBR-based microcavities.

We note that in ref. ^29^, a similar level attraction and inverted dispersion was observed for the trion-polariton branch at a large trion-photon detuning. However, their qualitative and heuristic model supporting this finding is not applicable in samples without large doping. We expect doping to be low in our as-exfoliated monolayer integrated into the high-quality flip-chip cavity, as presented previously^33^, where an anomalous dispersion is also observed at the exciton energy (see Supplementary Note 7). In contrast to ref. ^29^, our approach explains all observed features by incorporating exciton–phonon coupling and cavity losses, which are always present in these TMDC microcavities and are determined from ab initio calculations. As a result, we would expect that the joint action of cavity losses and exciton–phonon scattering should also contribute to the experimental scenario of ref. ^29^, and an effective non-Hermitian Hamiltonian with dissipative coupling could be an appropriate model.

Various types of dissipative coupling of different origins have been observed in optomechanical^12^ and magnon–photon systems^14^, and open microwave cavities^13,15^. It also appears in theoretical studies of fragmented exciton–polariton condensates^34^ and excitons polaritons coupled to an optomechanical resonator^35^. Similarly to cavity magnonics and optomechanics, dissipative coupling might be ubiquitous in exciton–photon systems and hence, the anomalous dispersion observed here could potentially also be observed in other semiconductor microcavities^36^, e.g., with III/V-semiconductors^24^, perovskites^10,37^, and organic semiconductors^38^.

Our work extends the arsenal of dispersion engineering tools for hybrid light-matter particles beyond the application of periodic fields and spin–orbit coupling^39,40^ by adding the possibility of novel, non-Hermitian disperison engineering. Here, we only showed the dynamics induced by negative *m*_1_. However, the anomalous dispersion can be further employed to demonstrate nontrivial wavepacket dynamics^25^ and negative-mass hydrodynamics^39^ due to both negative *m*_1_ and *m*_2_. For example, the negative-mass polaritons are expected to accelerate in the direction opposite to an applied force. It would be interesting to study bosonic condensation of polaritons in the anomalous dispersion regime^41^, where the energy minimum around *k* = 0 (see Fig. 2c) is not the global one. The inverted dispersion can also eliminate instabilities of exciton–polariton condensates^26^, which can, for example, enable studies of the Kardar-Parisi-Zhang phase in quantum systems without the complexities of an underlying lattice structure^42^.

## Methods

### Sample fabrication

The DBRs used for the microcavities presented in the main text are grown by plasma-enhanced chemical vapour deposition (PECVD) and consist of (bottom) 17.5 and (top) 15.5 alternating quarter-wave stack of SiO_*x*_ and SiN_*x*_, as schematically shown in Fig. 3a. Further, the first half of the SiO_2_ cavity spacer is deposited via RF-sputtering and finished with atomic layer deposition (ALD). The monolayer is then mechanically transferred at 120 ^∘^C on top of the oxygen-plasma-treated DBR substrate to increase the bonding between the monolayer and the substrate. To protect the monolayer against further material deposition, a 80 nm thick layer of poly-methyl-methacrylate (PMMA) is spin-coated on top of the structure. Before depositing the top DBR via PECVD to complete the structure, the cavity thickness and hence, the cavity mode, is fine-tuned with an intermediate PECVD grown SiO_*x*_ layer. The two DBRs enable a Q-factor of above 10^3^ (see Supplementary Note 4). More details about the fabrication process are reported in ref. ^16^.

### Optical measurements

The microcavity is excited with a frequency doubled Nd:YAG laser source at *λ* = 532 nm (*E* ≈ 2.33 eV), which is tightly focused onto the sample surface with a infinity corrected Mitutoyo NIR objective (NA = 0.65). The PL is collected with an in-house built optical microscope, which allows for spatial filtering with a square edge-filter prior to momentum-resolved imaging. The momentum-resolved PL-spectra are recorded with an Andor Shamrock 500i spectrograph equipped with an Andor iXon 888 EMCCD camera.

## Supplementary information

Supplementary Information

## Data Availability

The data that support the findings of this study are available from the corresponding authors upon request.
