# Publisher Correction: Negative-mass exciton polaritons induced by dissipative light-matter coupling in an atomically thin semiconductor

**DOI:** 10.1038/s41467-023-36962-7

**Published:** 2023-03-02

**Authors:** M. Wurdack, T. Yun, M. Katzer, A. G. Truscott, A. Knorr, M. Selig, E. A. Ostrovskaya, E. Estrecho

**Affiliations:** 1 ARC Centre of Excellence in Future Low-Energy Electronics Technologies and Department of Quantum Science and Technology, Research School of Physics, The Australian National University, Canberra, ACT 2601 Australia; 2 Department of Materials Science and Engineering, Monash University, Clayton, VIC 3800 Australia; 3 Songshan Lake Materials Laboratory, Dongguan, 523808 Guangdong China; 4 Institute of Physics, Chinese Academy of Science, 100190 Beijing, China; 5 Nichtlineare Optik und Quantenelektronik, Institut für Theoretische Physik, Technische Universität Berlin, 10623 Berlin, Germany; 6 Department of Quantum Science and Technology, Research School of Physics, The Australian National University, Canberra, ACT 2601 Australia

**Keywords:** Two-dimensional materials, Polaritons

Correction to: *Nature Communications* 10.1038/s41467-023-36618-6, published online 23 February 2023

In the original version of this Article, an acknowledgment sentence was erroneously inserted in the last paragraph of page 3. The sentence read ‘(The fabrication of the high-reflectivity substrate was performed at the ACT OptoFab node of the Australian National Fabrication Facility—a company established under the National Collaborative Research Infrastructure Strategy to provide nano and microfabrication facilities for Australia’s researchers.)’. This sentence has now been removed.

One sentence in the Acknowledgments section, which read ‘We acknowledge the Australian National Fabrication Facility (ANFF) OptoFab at its node in the Australian Capital Territory (ACT) for fabricating the high-quality DBR substrate, and the ANFF ACT node for the technical support in sample fabrication.’, has been replaced with the following: ‘We acknowledge the Australian National Fabrication Facility (ANFF) OptoFab, a company established under the National Collaborative Research Infrastructure Strategy to provide nano and microfabrication facilities for Australia’s researchers, for fabricating the high-quality DBR substrate, and the ANFF ACT node for the technical support in sample fabrication.’

This has now been corrected in the PDF and HTML versions of the Article.

