# Supplementary material for: Negative-mass exciton polaritons induced by dissipative light-matter coupling in an atomically thin semiconductor
Source: Nat Commun. 2023 Feb 23;14:1026. doi: 10.1038/s41467-023-36618-6 (PMC9950362; doi:10.1038/s41467-023-36618-6)
Supplement: Supplementary file 1 — Supplementary Information [file 41467_2023_36618_MOESM1_ESM.pdf]

# Supplementary Information:
# Negative-mass exciton polaritons induced by dissipative light-matter coupling in an atomically thin semiconductor

M. Wurdack,[1,*] T. Yun,[1,2,3,4] M. Katzer,[5] A. G. Truscott,[6] A. Knorr,[5] M. Selig,[5] E. A. Ostrovskaya,[1,†] and E. Estrecho[1,‡]

[1]*ARC Centre of Excellence in Future Low-Energy Electronics Technologies and Department of Quantum Science and Technology, Research School of Physics, The Australian National University, Canberra, ACT 2601, Australia*
[2]*Department of Materials Science and Engineering, Monash University, Clayton, Victoria, 3800, Australia*
[3]*Songshan Lake Materials Laboratory, Dongguan 523808, Guangdong, China*
[4]*Institute of Physics, Chinese Academy of Science, Beijing 100190, China*
[5]*Nichtlineare Optik und Quantenelektronik, Institut für Theoretische Physik, Technische Universität Berlin, 10623 Berlin, Germany*
[6]*Department of Quantum Science and Technology, Research School of Physics, The Australian National University, Canberra, ACT 2601, Australia*

## SUPPLEMENTARY NOTE 1: MICROSCOPICAL CALCULATIONS OF THE POLARITON DISPERSION

In order to self-consistently compute both the polariton dispersion and dephasing, we consider a microscopic Hamiltonian, containing excitons ($X$), phonons ($b$), as well as resonator ($C$) and reservoir photons ($D$), including their individual coupling

$$H = H_0 + H_{X-Phon} + H_{X-Phot} + H_{Phot-Phot}, \quad (1)$$

Here, the free contributions of the different species read $H_0 = \sum_{\mathbf{Q}_\parallel \sigma} E^X_{\mathbf{Q}_\parallel} X^{\dagger\sigma}_{\mathbf{Q}_\parallel} X^\sigma_{\mathbf{Q}_\parallel} + \sum_{\mathbf{K}_\parallel,\alpha} \hbar\omega^\alpha_{\mathbf{K}_\parallel} b^{\dagger\alpha}_{\mathbf{K}_\parallel} b^\alpha_{\mathbf{K}_\parallel} + \sum_{\bar{\mathbf{Q}}_\parallel,\sigma} E^C_{\bar{\mathbf{Q}}_\parallel} C^{\dagger\sigma}_{\bar{\mathbf{Q}}_\parallel} C^\sigma_{\bar{\mathbf{Q}}_\parallel} + \sum_{\mathbf{Q},\tau} E^D_{\mathbf{Q}} D^{\dagger\tau}_{\mathbf{Q}} D^\tau_{\mathbf{Q}}$, where the first term accounts for the dispersion of excitons $E^X_{\mathbf{Q}_\parallel} = E^X_0 + \frac{\hbar^2 \mathbf{Q}^2_\parallel}{2M}$ with the spectral energy $E^X_0$, the mass $M$ [1] of the exciton and the two dimensional center of mass momentum $\mathbf{Q}_\parallel$. $X^{(\dagger)\sigma}_{\mathbf{Q}_\parallel}$ denote exciton annihilation (creation) operators with the valley $\sigma = K, K' = +, -$, which we assume to commute as bosons in the zero density limit. The second term of $H_0$ denotes the phonon dispersion $\hbar\omega^\alpha_{\mathbf{K}_\parallel}$ obtained from DFT calculations [2, 3]. Furthermore $b^{(\dagger)\alpha}_{\mathbf{K}_\parallel}$ denote phonon annihilation (creation) operators. The third term accounts for the dispersion of the photons in the cavity $E^C_{\bar{\mathbf{Q}}_\parallel} = \sqrt{\left(E^C_0\right)^2 + \frac{\hbar^2 c^2 \bar{\mathbf{Q}}^2_\parallel}{(n^C)^2}}$, with the confinement energy $E^C_0 = \frac{\hbar c}{2 n^C d^C}$ adjusted to the experiment (see Supplementary Note 5), the speed of light $c$, the cavity refractive index $n^C$ and the cavity length $d^C$. Photon annihilation (creation) operators in the cavity are denoted by $C^{(\dagger)\sigma}_{\bar{\mathbf{Q}}_\parallel}$ with the photon polarization $\sigma = \sigma_+, \sigma_- = +, -$. The last term describes the dispersion of the free photons outside of the cavity $E^D_{\mathbf{Q}} = \frac{\hbar c|\mathbf{Q}|}{n^D}$.

The exciton-phonon interaction Hamiltonian is given as $H_{X-Phon} = \sum_{\mathbf{Q}_\parallel,\mathbf{K}_\parallel,\alpha,\sigma} g^\alpha_{\mathbf{K}_\parallel} X^{\dagger\sigma}_{\mathbf{Q}_\parallel+\mathbf{K}_\parallel} X^\sigma_{\mathbf{Q}_\parallel} \left(b^\alpha_{\mathbf{K}_\parallel} + b^{\dagger\alpha}_{-\mathbf{K}_\parallel}\right)$ with the exciton-phonon matrix element $g^\alpha_{\mathbf{K}_\parallel}$ [2–4]. The exciton-photon coupling Hamiltonian in rotating wave approximation with the excitonic dipole moment $\mathbf{d}$ [5, 6] is given as $H_{X-Phot} = \sum_{\mathbf{Q}_\parallel,\sigma} V_{\mathbf{Q}_\parallel} \left(X^{\dagger\sigma}_{-\mathbf{Q}_\parallel} C^\sigma_{-\mathbf{Q}_\parallel} + X^\sigma_{\mathbf{Q}_\parallel} C^{\dagger\sigma}_{\mathbf{Q}_\parallel}\right)$.

The appearing exciton-photon matrix element reads $V_{\mathbf{Q}_\parallel} = \sqrt{\frac{E^L_{\mathbf{Q}_\parallel}}{\epsilon_0 I^3}} \sin(\frac{\pi z}{d}) \mathbf{e}^\sigma \cdot \mathbf{d}$ (the appearing $\delta_{\mathbf{Q}_\parallel,\bar{\mathbf{Q}}_\parallel}$ was already accounted for). In order to describe the out-coupling of cavity photons to the vacuum, we write the phenomenolgical tunneling Hamiltonian $H_{Phot-Phot} = \sum_{\mathbf{Q}\sigma\tau} T^{\sigma\tau}_{\mathbf{Q}} \left(C^\sigma_{\bar{\mathbf{Q}}_\parallel} D^{\dagger\tau}_{\mathbf{Q}} + C^{\dagger\sigma}_{-\bar{\mathbf{Q}}_\parallel} D^\tau_{-\mathbf{Q}}\right)$. The Heisenberg equation of motion formalism allows to find a closed set of linear equations, namely the equations of motion (EOM) for the coherent exciton amplitude $\langle X^\sigma_{\mathbf{Q}_\parallel}\rangle$, the coherent cavity photon amplitude $\langle C^\sigma_{\bar{\mathbf{Q}}_\parallel}\rangle$ and the coherent amplitude of the free photons outside the cavity $\langle D^\tau_{\mathbf{Q}}\rangle$. To close the system, it is necessary to also compute equations of the respective phonon assisted amplitudes $\langle X^\sigma_{\mathbf{Q}_\parallel} b^{(\dagger)\alpha}_{\mathbf{K}_\parallel}\rangle$, $\langle C^\sigma_{\bar{\mathbf{Q}}_\parallel} b^{(\dagger)\alpha}_{\mathbf{K}_\parallel}\rangle$ and $\langle D^\tau_{\mathbf{Q}} b^{(\dagger)\alpha}_{\mathbf{K}_\parallel}\rangle$. Reflecting the strong coupling between excitons and cavity photons, the whole set of EOM is diagonalised with respect to the coupling $M_{\mathbf{Q}_\parallel}$, which gives $X^\sigma_{\mathbf{Q}_\parallel} = \alpha_{\mathbf{Q}_\parallel} P^-_{\mathbf{Q}_\parallel\sigma} + \beta_{\mathbf{Q}_\parallel} P^+_{\mathbf{Q}_\parallel\sigma}$, and $C^\sigma_{\mathbf{Q}_\parallel} = \alpha_{\mathbf{Q}_\parallel} P^+_{\mathbf{Q}_\parallel\sigma} - \beta_{\mathbf{Q}_\parallel} P^-_{\mathbf{Q}_\parallel\sigma}$, with the coherent amplitudes of the (upper and lower) polariton branches $P^\pm_{\mathbf{Q}_\parallel\sigma}$ and the complex Hopfield coefficients $\alpha_{\mathbf{Q}_\parallel}, \beta_{\mathbf{Q}_\parallel} = \frac{1}{\sqrt{2}}\sqrt{1 \pm \frac{\tilde{\Delta}_{\mathbf{Q}_\parallel}}{\sqrt{\tilde{\Delta}^2_{\mathbf{Q}_\parallel} + 4\tilde{V}^2_{\mathbf{Q}_\parallel}}}}$, with the energy detuning $\tilde{\Delta}_{\mathbf{Q}_\parallel} = E^C_{\mathbf{Q}_\parallel} - E^X_{\mathbf{Q}_\parallel} - i\big(\gamma^C_{\mathbf{Q}_\parallel} - \gamma^X_{\mathbf{Q}_\parallel}\big)$ and the complex coupling between excitons and cavity photons $\tilde{V}_{\mathbf{Q}_\parallel} = V_{\mathbf{Q}_\parallel} + i g_{\mathbf{Q}_\parallel}$. Note that both $\tilde{\Delta}_{\mathbf{Q}_\parallel}$ and $\tilde{V}_{\mathbf{Q}_\parallel}$ are complex terms, with the real parts stemming directly from the Hamiltonians for dispersion and exciton-photon interaction, respectively. The imaginary parts are however to be found as the solution vector $(\gamma^X_{\mathbf{Q}_\parallel}, \gamma^C_{\mathbf{Q}_\parallel}, g_{\mathbf{Q}_\parallel})$ of the self consistent EOM, as will be shown in the following. After diagonalisation, the EOM of the phonon assisted quantities can be solved subsequently in a Born-Markov approach, (for $\langle X^\sigma_{\mathbf{Q}_\parallel} b^{(\dagger)\alpha}_{\mathbf{K}_\parallel}\rangle$ even a second diagonalisation is necessary). Eventually, we

end up with

$$\Big(i\hbar\partial_t - E^{+}_{\mathbf{Q}_\parallel} + i\alpha^2_{\mathbf{Q}_\parallel}\Gamma^{Pt+}_{\mathbf{Q}_\parallel} + i\beta^2_{\mathbf{Q}_\parallel}\Gamma^{Pn+}_{\mathbf{Q}_\parallel}\Big)\langle P^{+}_{\mathbf{Q}_\parallel\sigma}\rangle - i\alpha_{\mathbf{Q}_\parallel}\beta_{\mathbf{Q}_\parallel}\big(\Gamma^{Pt-}_{\mathbf{Q}_\parallel} - \Gamma^{Pn-}_{\mathbf{Q}_\parallel}\big)\langle P^{-}_{\mathbf{Q}_\parallel\sigma}\rangle = \beta_{\mathbf{Q}_\parallel}\Omega^{\sigma}_{\mathbf{Q}_\parallel} \quad (2)$$

$$\Big(i\hbar\partial_t - E^{-}_{\mathbf{Q}_\parallel} + i\beta^2_{\mathbf{Q}_\parallel}\Gamma^{Pt-}_{\mathbf{Q}_\parallel} + i\alpha^2_{\mathbf{Q}_\parallel}\Gamma^{Pn-}_{\mathbf{Q}_\parallel}\Big)\langle P^{-}_{\mathbf{Q}_\parallel\sigma}\rangle - i\alpha_{\mathbf{Q}_\parallel}\beta_{\mathbf{Q}_\parallel}\big(\Gamma^{Pt+}_{\mathbf{Q}_\parallel} - \Gamma^{Pn+}_{\mathbf{Q}_\parallel}\big)\langle P^{+}_{\mathbf{Q}_\parallel\sigma}\rangle = \alpha_{\mathbf{Q}_\parallel}\Omega^{\sigma}_{\mathbf{Q}_\parallel}. \quad (3)$$

Where $\Omega^{\sigma}_{\mathbf{Q}_\parallel}$ accounts for the external driving, and the microscopically derived dephasing due to the photonic reservoir outside the cavity reads $\Gamma^{Pt\pm}_{\mathbf{Q}_\parallel} = \pi\sum_{Q_z,\tau\sigma}(T^{\sigma\tau}_{\mathbf{Q}})^2\delta(E^{D}_{\mathbf{Q}} - E^{\pm}_{\mathbf{Q}_\parallel})$ and the derived dephasing due to the phonons in the atomically thin transition metal dichalcogenide crystal (TMDC) can be written as $\Gamma^{Pn\pm}_{\mathbf{Q}_\parallel} = \pi\sum_{\mathbf{K}_\parallel,\alpha}|g^{\alpha}_{|\mathbf{K}_\parallel-\mathbf{Q}_\parallel|}|^2\Big(\bar{\alpha}^2_{\mathbf{K}_\parallel}(1+n^{\alpha}_{|\mathbf{K}_\parallel-\mathbf{Q}_\parallel|})\delta(\mathcal{E}^{L}_{\mathbf{K}_\parallel} - E^{\pm}_{\mathbf{Q}_\parallel} + \hbar\omega^{\alpha}_{|\mathbf{K}_\parallel-\mathbf{Q}_\parallel|}) + \bar{\alpha}^2_{\mathbf{K}_\parallel}n^{\alpha}_{|\mathbf{K}_\parallel-\mathbf{Q}_\parallel|}\delta(\mathcal{E}^{L}_{\mathbf{K}_\parallel} - E^{\pm}_{\mathbf{Q}_\parallel} - \hbar\omega^{\alpha}_{|\mathbf{K}_\parallel-\mathbf{Q}_\parallel|}) + \bar{\beta}^2_{\mathbf{K}_\parallel}(1+n^{\alpha}_{|\mathbf{K}_\parallel-\mathbf{Q}_\parallel|})\delta(\mathcal{E}^{U}_{\mathbf{K}_\parallel} - E^{\pm}_{\mathbf{Q}_\parallel} + \hbar\omega^{\alpha}_{|\mathbf{K}_\parallel-\mathbf{Q}_\parallel|}) + \bar{\beta}^2_{\mathbf{K}_\parallel}n^{\alpha}_{|\mathbf{K}_\parallel-\mathbf{Q}_\parallel|}\delta(\mathcal{E}^{U}_{\mathbf{K}_\parallel} - E^{\pm}_{\mathbf{Q}_\parallel} - \hbar\omega^{\alpha}_{|\mathbf{K}_\parallel-\mathbf{Q}_\parallel|})\Big)$, with the phonon occupation $n^{\alpha}_{\mathbf{Q}_\parallel} = \langle b^{\dagger\alpha}_{\mathbf{Q}_\parallel}b^{\alpha}_{\mathbf{Q}_\parallel}\rangle$ computed from a Bose-Einstein statistics for the lattice temperature. We stress that for both the photonic and the phononic dispersion channels, the Fermi-rules in the delta functions secure that all scattering events obey energy conservation with respect to the self-consistently computed exciton-polariton dispersion $E^{\pm}_{\mathbf{Q}_\parallel} = \frac{E^X_{\mathbf{Q}_\parallel}+E^C_{\mathbf{Q}_\parallel}}{2} \pm \mathfrak{Re}\left(\sqrt{\frac{\tilde{\Delta}^2_{\mathbf{Q}_\parallel}}{4} + \tilde{V}^2_{\mathbf{Q}_\parallel}}\right)$. (The imaginary counterpart of this dispersion gives the respective dephasing in the diagonalised basis, $\gamma^{\pm}_{\mathbf{Q}_\parallel} = \frac{\gamma^X_{\mathbf{Q}_\parallel}+\gamma^C_{\mathbf{Q}_\parallel}}{2} \mp \mathfrak{Im}\left(\sqrt{\frac{\tilde{\Delta}^2_{\mathbf{Q}_\parallel}}{4} + \tilde{V}^2_{\mathbf{Q}_\parallel}}\right)$.) Eq. (2,3) can be mapped on a phenomenological model, where $\langle P^{+}_{\mathbf{Q}_\parallel\sigma}\rangle$ and $\langle P^{-}_{\mathbf{Q}_\parallel\sigma}\rangle$ must be decoupled after diagonalisation, which gives in total four equations for the computation of the dephasing

$$\gamma^{+}_{\mathbf{Q}_\parallel} = \alpha^2_{\mathbf{Q}_\parallel}\Gamma^{Pt+}_{\mathbf{Q}_\parallel} + \beta^2_{\mathbf{Q}_\parallel}\Gamma^{Pn+}_{\mathbf{Q}_\parallel} \quad (4)$$

$$\gamma^{-}_{\mathbf{Q}_\parallel} = \beta^2_{\mathbf{Q}_\parallel}\Gamma^{Pt-}_{\mathbf{Q}_\parallel} + \alpha^2_{\mathbf{Q}_\parallel}\Gamma^{Pn-}_{\mathbf{Q}_\parallel} \quad (5)$$

$$0 = \alpha_{\mathbf{Q}_\parallel}\beta_{\mathbf{Q}_\parallel}\big(\Gamma^{Pt-}_{\mathbf{Q}_\parallel} - \Gamma^{Pn-}_{\mathbf{Q}_\parallel}\big) \quad (6)$$

$$0 = \alpha_{\mathbf{Q}_\parallel}\beta_{\mathbf{Q}_\parallel}\big(\Gamma^{Pt+}_{\mathbf{Q}_\parallel} - \Gamma^{Pn+}_{\mathbf{Q}_\parallel}\big) \quad (7)$$

This set of equations completely determines the dephasing, which means that its solution gives the dephasing also in the original basis, i.e. $\gamma^X_{\mathbf{Q}_\parallel}$ as the dephasing of the excitons, $\gamma^C_{\mathbf{Q}_\parallel}$ as the respective dephasing of the cavity photons, and a third dephasing $g_{\mathbf{Q}_\parallel}$, which corresponds to the imaginary part of $\tilde{V}_{\mathbf{Q}_\parallel}$ and is off-diagonal in the original basis of the Hamiltonian. Those three parts of the dephasing constitute a vector of numerical solutions of Eqs. (4-7). Since $\Gamma^{Pt\pm}_{\mathbf{Q}_\parallel} = \Gamma^{Pn\pm}_{\mathbf{Q}_\parallel}$ is unlikely, we reduce Eqs. (6,7) to the more likely scenario $\alpha_{\mathbf{Q}_\parallel}\beta_{\mathbf{Q}_\parallel} = 0$, which reduces the set of equations to match the dimension of the solution vector $(\gamma^X_{\mathbf{Q}_\parallel}, \gamma^C_{\mathbf{Q}_\parallel}, g_{\mathbf{Q}_\parallel})$. As exciton-phonon scattering occurs at a range of momenta and energies substantially exceeding that of the light-cone and the light-matter coupling region, $\mathbf{K}_\parallel \gg \mathbf{Q}_\parallel$, we approximate $\mathbf{Q}_\parallel \approx 0$ and $|\mathbf{K}_\parallel - \mathbf{Q}_\parallel| \approx \mathbf{K}_\parallel$ [7], which significantly reduces the computational overhead. This leaves us with the solution vector $(\gamma^X_0, \gamma^C_0, g_0)$.

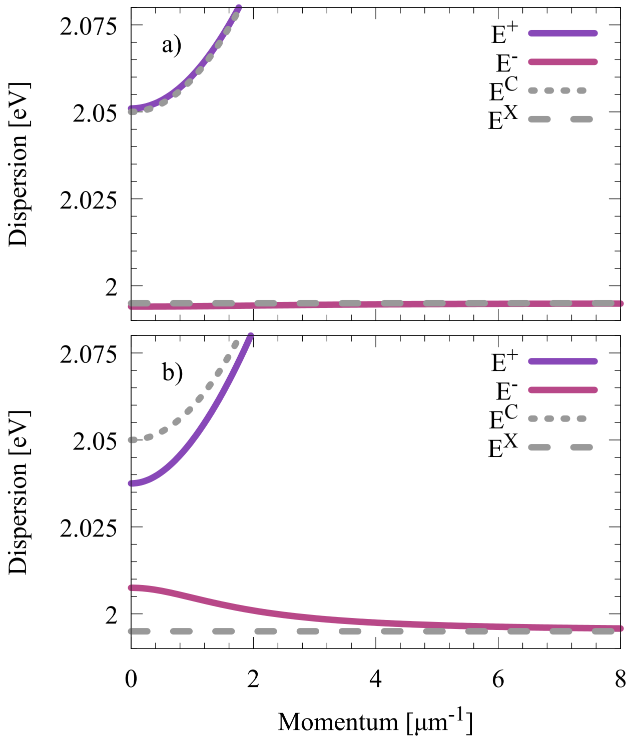


SUPPLEMENTARY FIGURE 1. Calculated dispersion of (red) the upper ($E^+$) and lower ($E^-$) polariton branches, and (black dashed) the dispersion of the uncoupled excitons, $E^X$, and of the cavity photons, $E^C$. **a**, Solution without the phonon bath, **b**, solution with the phonon bath. The level attraction can be traced back to the off-diagonal dephasing $g_{\mathbf{Q}_\parallel}$, which is computed self consistently from the interplay of phononic dephasing in the TMDC and photonic dephasing from the cavity loss.

Solving this system of equations with $V_0 = 8.3\,\mathrm{meV}$, the exciton-photon coupling strength for $Q_\parallel = 0$, $E^C_0 = 2.05\,\mathrm{eV}$, $E^X_0 = 1.995\,\mathrm{eV}$, and $T_0 = 0.54\,\mathrm{eV}$ (corresponding to a cavity linewidth of $\Gamma_C = T_0^2/\hbar c_0 \approx 1.47\,\mathrm{meV}$), we find an off-diagonal dephasing of $g_0 \approx 23\,\mathrm{meV}$, which significantly exceeds its real valued counterpart $V_0$, as it is assumed correctly in the phenomenological model in the main text. The microscopically calculated photonic and excitonic dephasings are $\gamma^C_0 \approx 1.8\,\mathrm{meV}$ and $\gamma^X_0 \approx 22\,\mathrm{meV}$, respectively.

Note, that these solutions are not directly related to the measured linewidths of the uncoupled systems. In particular, $\gamma^C_0$ differs from the cavity linewidth $\Gamma_C$ measured without the presence of the TMDC, and $\gamma_X$ differs from the value one would expect for a TMDC without a cavity surrounding. Both are altered by the strong coupling, and cannot be interpreted

without the third dissipation factor arising in this strong coupling regime, which we call off-diagonal dephasing $g_0$. This term relates to the terms $g_c$ and $g_x$ used in the phenomenological model, which represents the dissipative coupling via the photon and phonon baths, respectively. Note however that its value contains contributions from all elements in the $W$ matrix in our phenomenological model.

Supplementary Figure 1 shows the dispersion for the numerically computed values. It is evident that the level attraction is absent when not taking phonons into account (panel a), while with phonons it becomes clearly visible (panel b). This further supports our hypothesis that it is the exciton-phonon coupling that mainly leads to level attraction in our experiment and that the dissipative coupling via the phonon bath is the dominant channel in this material system. Hence, in the phenomenological model, $g_c \ll g_x$, as we used in the main text.

It is important to stress that this microscopic model does not contain any fitting parameters; all applied values rely on ab initio calculations from the literature [1–3, 5]. Since a coupled oscillator Hamiltonian can only produce level attraction (as shown in Supplementary Figure 1b) when accounting for off-diagonal dephasing, our microscopic model justifies the parameters $g_c$ and $g_x$ in the phenomenological model, Eqs. (1,2) in the main text. Therefore, the theory yields a good qualitative agreement between our microscopic calculations, our phenomenological Hamiltonian, and the experiment, with both dissipative coupling and level attraction present. Reproducing the experimentally observed maximum two-level attraction at $k \neq 0$ and formally deriving the phenomenological Hamiltonian from the microscopic model is subject to further studies.

## SUPPLEMENTARY NOTE 2: ENERGY LEVEL DYNAMICS IN $\Delta$-$V$ PARAMETER SPACE

The general behaviour of the energy eigenvalues presented in the main text is further described here. Similar to the main text, we apply the approximation $\gamma_c << \gamma_x$, which holds for TMDs embedded in all-dielectric microcavities at room temperature [8, 9], and thus, set $g_x = g$ and $g_c = 0$ Supplementary Figure 2a shows the mean-subtracted energies $E - \langle E \rangle$ in the $\Delta$-$V$ parameter space, highlighting the position of the non-Hermitian degeneracy, the exceptional point. Without dissipative coupling, i.e. when $g = 0$, the exceptional point occurs at resonance $\Delta = 0$ with the critical coherent coupling strength $V_c = |(\gamma_x - \gamma_c)|/2$. The dissipative coupling term $g$ shifts the exceptional point away from resonance towards $\Delta = 2\sqrt{g\gamma_x}$, while counter-intuitively reducing the critical coherent coupling strength to $V_c = |(\gamma_x - \gamma_c - g)|/2$. This is because $\gamma_c$ is significantly smaller than $\gamma_x$ in these TMD microcavities, so $g$ fills in the difference, resulting in a smaller $V_c$.

Supplementary Figure 2b shows the energy deviation $\Delta_{UP} = E_U - E_L - \Delta$ as a function of $\Delta$ and $V$ for $g = 0.4\gamma_x$.

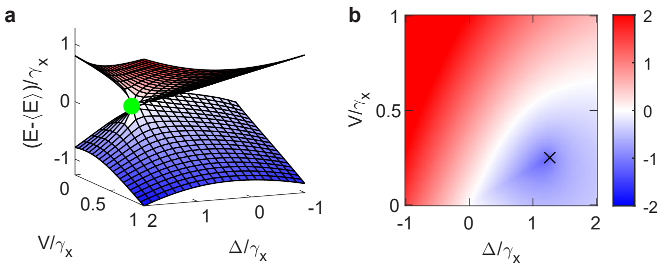


SUPPLEMENTARY FIGURE 2. **Exceptional point and energy deviation.** **a**, Mean-subtracted energy surfaces of the eigenvalue branches, and **b**, energy deviation $\Delta_{UP}$ in the $\Delta$-$V$ parameter space. The exceptional point (green dot and '×' mark) is at $V = |\gamma_x - \gamma_c - g|/2$, $\Delta = 2\sqrt{g\gamma_x}$. Other parameters are: $\gamma_c = 0.1\gamma_x$ and $g = 0.4\gamma_x$.

Regions in red (blue) represent level repulsion (attraction), and the white region represent the transition between them. This clearly shows that level attraction will only become apparent when $V$ is sufficiently small. It is also important to stress that maximum level attraction (negative $\Delta_{UP}$) occurs at the exceptional point, which is marked by '×' in Supplementary Figure 2b. This is because the exceptional point (where the coupled energies cross) is shifted away from $\Delta = 0$ (where the uncoupled energies cross), resulting in a large energy difference between the coupled and uncoupled energies.

## SUPPLEMENTARY NOTE 3: DISPERSIONS AT ZERO AND NEGATIVE EXCITON–PHOTON DETUNINGS

Supplementary Figure 3 shows supplementary exciton–photon dispersions at different detunings $\Delta_0$ and coupling strengths $V$ and $g$. The case for resonant ($\Delta_0 = 0$) detuning is shown in Supplementary Figure 3a, which shows similar features as those in the main text, i.e. an inverted dispersion at finite $k$, when the dissipative coupling strength $g$ is strong. The same features are exhibited by the negative detuning case in Supplementary Figure 3b, except for the crossing in energies when $V = 0$, which is expected in the weak coupling regime.

The interesting case of a very positive detuning, with $\Delta_0 = 2\gamma_x$, is shown in Supplementary Figure 3c, which features an inverted dispersion centred at $k = 0$, similar to Supplementary Figure 1b. Here, both $m_1$ and $m_2$ are negative around $k = 0$ with $m_1$ being negative for a wide range of momenta $\hbar k$. Note that in the two cases with $g = 0.6\gamma_x$, the lower branch is blueshifted from the exciton line. The parameters here correspond to the upper region of Supplementary Figure 2f of the main text.

We also show the dispersion for the set of parameters where there is no negative $m_1$, as presented in Supplementary Figure 3d, corresponding to the upper and lower regions in Supplementary Figure 2d and 2e of the main text, respectively. Examples are given for the case when $g$ is present but weak, and when $g$ is strong but $V$ is stronger. In both cases at pos-

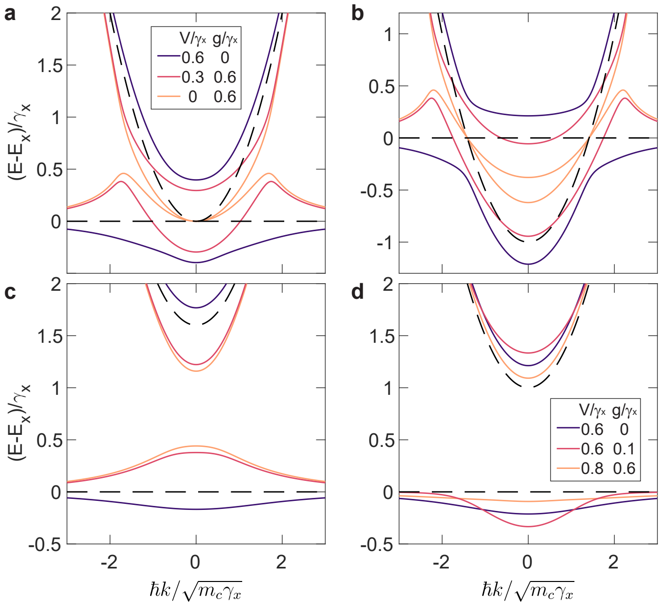


SUPPLEMENTARY FIGURE 3. **Dispersions at different exciton–photon detunings. a,** Zero ($\Delta_0 = 0$), **b,** negative ($\Delta_0 = -\gamma_x$), and **c,** highly positive ($\Delta_0 = 2\gamma_x$) detunings. Dashed lines correspond to the exciton and cavity photon dispersions. The values for $V$ and $g$ in **b** and **c** are the same as those in **a**.

itive detuning, the lower branch remains below the exciton line.

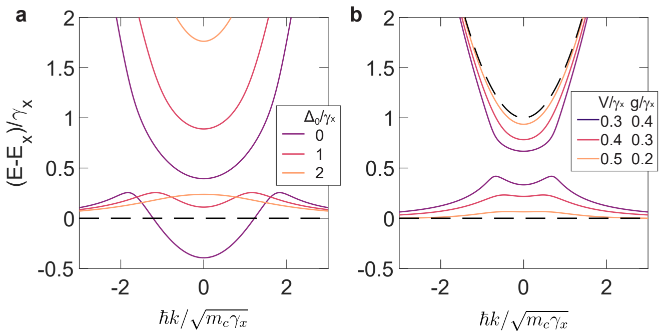


SUPPLEMENTARY FIGURE 4. **Inverted dispersion for different $\Delta_0$, $V$ and $g$. a,** Fixed $V = 0.4\gamma_x$ and $g = 0.6\gamma_x$. **b,** Fixed $\Delta_0 = \gamma_x$. Dashed lines correspond to the exciton and cavity photon dispersions. Bare cavity dispersions not shown in **a** for clarity. The values (from top to bottom of the legend) of $\Delta_0/(2\sqrt{g\gamma_x})$ are (**a**) $[0, 0.65, 1.29]$ and (**b**) $[0.79, 0.91, 1.12]$.

Supplementary Figure 4a shows the inverted dispersion as detuning is tuned at fixed $V$ and $g$. One can clearly see that the inverted peak at finite $k$ moves to $k = 0$ at high detuning $\Delta_0$, as shown by the map in Supplementary Figure 2f of the main text and by Supplementary Figure 3a-c. A similar behaviour occurs if we fix $\Delta_0$ but simultaneously tune the relative strengths of $V$ and $g$. As shown in Supplementary Figure 4b, as the $V$ increases and $g$ decreases, the inversion peak moves towards $k = 0$. This behaviour corresponds to the difference in dispersion of the two samples shown in the main text (Supplementary Figure 3b,c), and what we think occurs when we compare our samples with degraded oscillator strength (see Supplementary Figure 3c of the main text) to those with high oscillator strengths (see Supplementary Figure 10 and discussion in Supplementary Note 9).

## SUPPLEMENTARY NOTE 4: OPTICAL CHARACTERISATION OF THE $WS_2$ EXCITONS AND THE MICROCAVITY PHOTONS OF THE STRUCTURE PRESENTED IN THE MAIN TEXT.

The structure investigated in the main text was fabricated with the method presented in Ref. [9]. In contrast to this work, we deposited the DBR substrate consisting of 17.5 $SiN_x/SiO_x$ layers on top of a silicon chip via plasma-enhanced chemical vapour deposition (PECVD) at 300 °C, and finalised it with 100 nm of $SiO_2$, of which 80 nm was grown via sputtering and 20 nm via atomic layer deposition (ALD). The $WS_2$ monolayer was then mechanically exfoliated [10] from bulk $WS_2$ sourced from HQ graphene [11] and placed onto the DBR substrate. As a reference, we placed a $WS_2$ monolayer onto a high-quality $SiO_2$ chip from Nova Materials [12].

After spin-coating the 80 nm thick PMMA cavity spacer and protective layer on both samples, we compare the effects of the substrate on the exciton-photon interactions in these monolayer by measuring the reflectivity spectra with a tungsten halogen white light source.

Supplementary Figure 5a presents the reflectivity contrast spectra $\Delta R/R_{Ref}$ of both samples. The spectra were calculated by measuring the reflectivty spectra on the monolayer $R$ and next to the monolayers $R_{Ref}$, with $\Delta R = R - R_{Ref}$. When fitting a Voigt-profile to the high-energy shoulder of the reflectivity spectrum of the monolayer placed on the DBR chip, we find for the exciton energy and linewidth $E_X \approx 2.0073$ eV and $\gamma_x = (62.5 \pm 0.2)$ meV, respectively, with a homogeneous linewidth of $\gamma_x^H = (34.8 \pm 0.7)$ meV and an inhomogeneous linewidth of $\gamma_x^I = (40.8 \pm 0.7)$ meV.

Here, the product of amplitude and homogeneous linewidth at the exciton energy scales with the exciton oscillator strength quantifying the exciton-photon interactions [13]. Clearly, the oscillator strength of the monolayer on the DBR chip is significantly lower compared to that of the reference sample. Therefore, the DBR substrate lowers the exciton oscillator strength of the monolayer compared to that when placed onto a high quality substrate, which is possibly due to environmental charge doping, dielectric disorder and strain [14–19].

Moreover, as we have demonstrated in Ref. [9], the exciton oscillator strength will further decrease after deposition of the top structure, consisting of the $SiO_x$ spacer and the top DBR, via PECVD at 150 °C. While a reduction of the exciton oscillator strength is normally avoided for polariton research, it amplifies the effects of dissipative coupling on the polariton dispersion and allows us in this work to observe the negative mass of the lower polariton branch. Additionally, the further

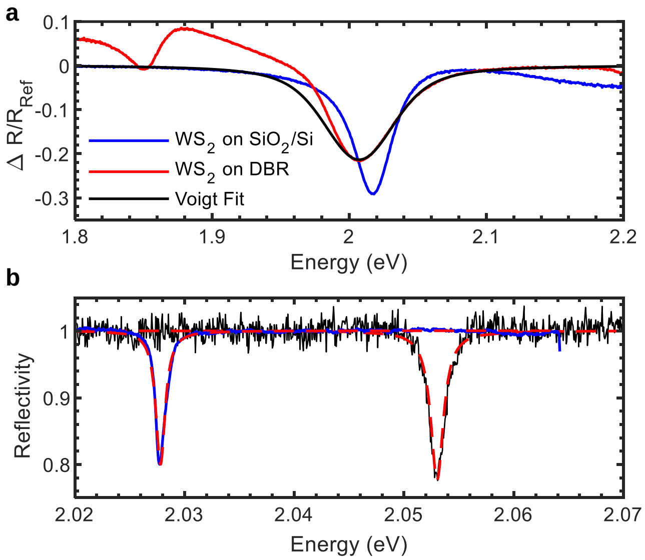


SUPPLEMENTARY FIGURE 5. **Characterisation of the microcavity discussed in the main text and the excitonic response of monolayer $WS_2$ on the DBR substrate. a**, The derivative of the reflectivity contrast spectrum of a $WS_2$ monolayer capped with PMMA on (blue) a high-quality $SiO_2$ substrate and (red) on the DBR chip. The reflectivity contrast spectrum was fitted with (black) a Voigt profile. **b**, Reflectivity spectra of the microcavities presented in the main text next to the monolayer regions, where (black solid line) is the spectrum of sample 1, (blue solid line) the spectrum of sample 2, and (red dashed line) Lorentzian fits to the respective spectra.

deposition of the top structure of the microcavitiy causes a red-shift of the exciton energy [9].

After finalising the two microcavities presented in the main text, we further measured the reflectivity spectra outside of the monolayer areas at zero momentum $k = 0$ to characterise the photon mode. Supplementary Figure 5b shows strong reflectivity dips at $E \approx 2.053$ meV and $E \approx 2.028$ meV marking the cavity resonances of the two samples $E_c$, which are positively detuned from the bare exciton energy. The linewidths of the cavity modes are $\gamma_c = 1.4$ meV for sample 1 and $\gamma_c = 1$ meV for sample 2 and therefore, the microcavities have Q-factors of around 1500 and 2000, respectively.

## SUPPLEMENTARY NOTE 5: PEAK ENERGIES OF THE ANGLE-RESOLVED PL OF THE LOADED MICROCAVITY

To extract the peak energies of the angle-resolved PL spectra of the monolayers embedded in the microcavity, we fit the spectra at each $k$ with a two-peak Voigt profile. Exemplary fitting results for the highly positively-detuned sample of the main text (see Supplementary Figure 3c) are presented in Supplementary Figure 6. Clearly, the peak energy of the lower branch reaches its maximum value at around $k = 2\ \mu\text{m}^{-1}$ and decreases at larger $k$ towards the exciton energy (dashed line). This behaviour demonstrates the maximum level attraction at finite $k$, as predicted by our model (see Supplementary Figure 1c and Fig2a of the main text), resulting in the anomalous dispersion and negative mass polaritons as presented in the main text (see Supplementary Figure 3c).

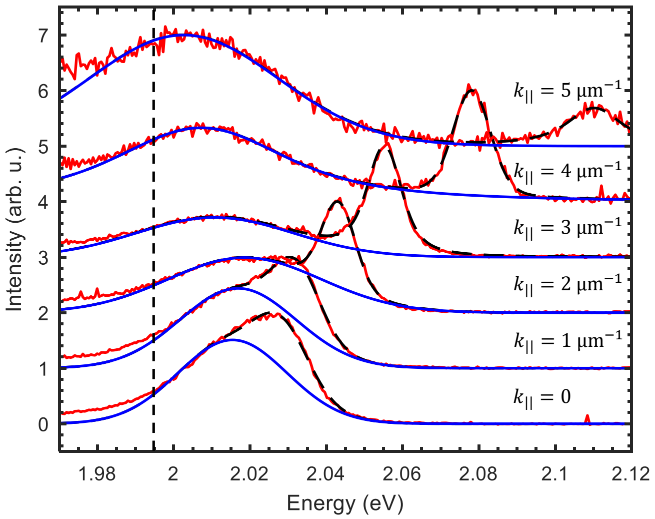


SUPPLEMENTARY FIGURE 6. **Waterfall plot of the angle-resolved PL spectrum at increasing momenta $k_{||}$.** The red lines are the extracted spectra at different $k$, the black dashed lines are the two-peak Voigt fit, and the blue lines are the contribution of the lower branch to the fits. For reference, the fitted exciton energy is marked at 1.995 eV. The data correspond to the highly positively detuned sample (see Supplementary Figure 3c of the main text).

## SUPPLEMENTARY NOTE 6: REFLECTANCE AND LOW TEMPERATURE MEASUREMENTS

We fabricated a third sample with the right size and cleaner surrounding areas to enable clear reflectance measurements of the anomalous dispersion. The angle-resolved reflectance contrast and PL spectra of this sample are presented in Supplementary Figure 7a,b. The negative-mass dispersion of the lower branch is visible at high momenta, $k > \pm 2.5\ \mu\text{m}^{-1}$ and energies below 2.03 eV. Clearly, the lower branch tends to redshift with increasing $k$ towards the exciton line around $\sim 1.99$ eV and $\sim 1.98$ eV for the reflectance and PL, respectively. This is the anomalous dispersion, which is due to the level attraction and hence strong dissipative coupling in this system.

The difference in the bare exciton energies between the reflectance contrast and PL measurements originates from the Stokes shift in this sample. This is shown in Supplementary Figure 7**c** which were measured on the monolayer on DBR before the top mirror deposition. Note that the further PECVD growth on the structure will redshift the exciton line and change the Stoke's shift as we previously demonstrated [20]. This explains the redshift of the exciton lines when the monolayer is fully embedded in the microcavity.

The anomalous dispersion observed in the reflectance con-

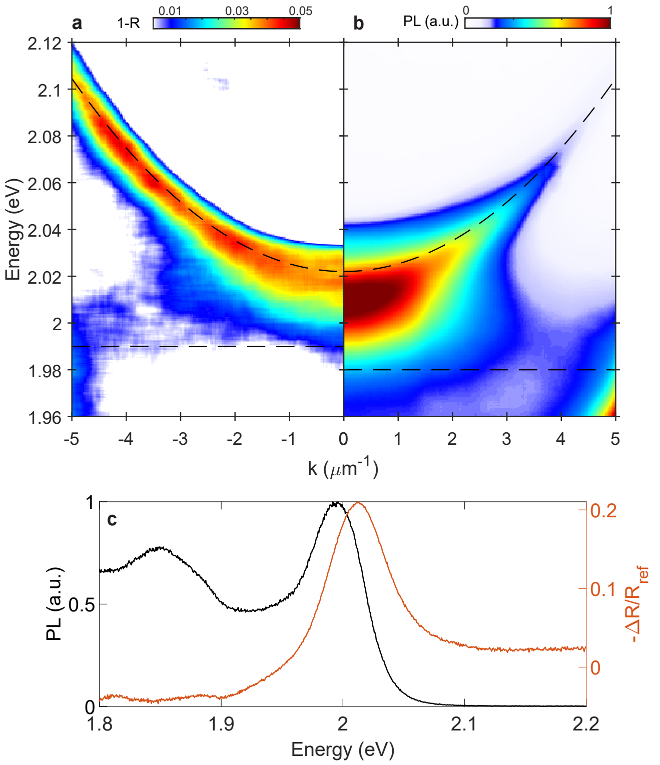


SUPPLEMENTARY FIGURE 7. **Characterisation of Sample 3 at room temperature.** Angle-resolved **a**, reflectance contrast and **b**, photoluminescence of a third sample. In **a**, $R = \Delta R/R_{ref}$. Dashed lines correspond to the bare exciton and cavity photon dispersion. **c**, PL and negative reflectance contrast $-\Delta R/R_{ref}$ of the monolayer on DBR (before top mirror deposition), showing a Stokes shift of $\approx$ 18 meV.

trast (or absorption) measurements further confirms that the anomalous dispersion observed in the PL measurements correspond to a mode with dissipative coupling and is not a result of excitonic-fraction dependent Stokes shift of the polariton emission, as it was proposed in previous work [21].

To demonstrate tunability on the same sample, we cooled down the third sample to liquid He temperature. At 4 K, the exciton line in our sample blueshifts by around $\sim$ 90 meV and its linewidth drastically narrows [22]. Furthermore, we expect the exciton-phonon coupling, and hence, the dissipative coupling to be much weaker compared to values at room temperature. Therefore, the level attraction would be diminished as the level repulsion (or coherent coupling) becomes dominant.

This is indeed what we observe in the angle-resolved PL spectra shown Supplementary Figure 8. The exciton-photon detuning at this temperature is very negative due to the strong blueshift of the exciton line $\sim$ 90 meV. A clear anti-crossing, and thus, level repulsion, is observed around $\sim$ 2.08 meV which is the main signature of strong coherent coupling between excitons and photons. Hence, we have transitioned from a system dominated by level attraction (dissipative coupling) to that dominated by level repulsion (coherent coupling) on the same sample by tuning the temperature. This is further evidence that the dissipative coupling observed in our samples is indeed a result of exciton-phonon scattering as derived in the microscopic theory (see Supplementary Note 1). A proper study of the effects of temperature on level attraction (or dissipative coupling) requires a sample, in which we can dynamically adjust the cavity energy while reducing the temperature to maintain a constant exciton-photon detuning. This can be done, for example, with an open cavity device, e.g., [21], and is subject to further studies.

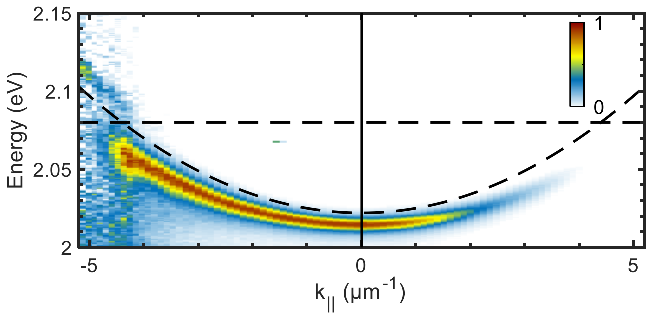


SUPPLEMENTARY FIGURE 8. **PL of Sample 3 at low temperature.** Normalised (left) and raw (right) angle-resolved PL spectra of Sample 2 at 4 K with the (black dashed line) uncoupled exciton and photon modes. A clear anti-crossing is observed at the crossing between the exciton and photon modes at $\sim$ 2.08 eV.

## SUPPLEMENTARY NOTE 7: COMPARISON WITH A FLIP-CHIP CAVITY WITH A HIGH-QUALITY DBR SUBSTRATE

As highlighted in the main text, the quenching of the coherent coupling term and the subsequent domination of the dissipative coupling term arises mainly from the microcavity fabrication process and the quality of the DBR. To support this postulate, we fabricated another sample, we call Sample 4, using a flip-chip approach [23] and a high-quality DBR substrate [24], see Supplementary Figure 9a. We have used this approach before to create high-quality exciton polaritons [8, 25] so we expect that coherent coupling is much stronger in this sample.

The key difference in this sample is the preservation of the large exciton oscillator strength of the monolayer. In comparison to the monolayer $WS_2$ on PECVD-grown DBR (see 5a), the monolayer $WS_2$ on top of the high-quality DBR maintains a large reflectance dip (see Supplementary Figure 9b), which is proportional to the oscillator strength or the coherent coupling strength. More importantly, this large value is unaffected by the top DBR chip since it is not deposited onto the monolayer [23]. This is unlike the PMMA-PECVD approach mainly used in this work, which unavoidably encapsulates the monolayer and exposes it to the plasma involved in the PECVD growth.

The resulting PL spectra of Sample 4 is presented in Sup-

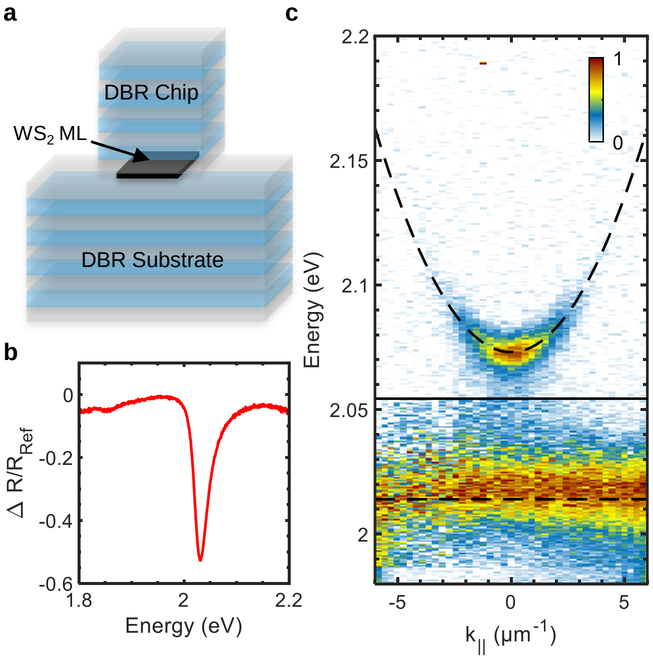


SUPPLEMENTARY FIGURE 9. **Flip-chip microcavity with a positive exciton-photon detuning.** **a**, Schematics of the microcavity design with an integrated monolayer (ML) $WS_2$ [23]. **b**, Normalized reflectivity spectrum of a monolayer $WS_2$ placed onto the high-quality DBR substrate [24]. **c**, Angle-resolved PL spectrum of the structure with the (black dashed lines) uncoupled exciton and photon modes.

plementary Figure 9c showing a well separated upper and lower branches due to the large positive exciton-photon detuning. Interestingly, the lower branch shows a small negative curvature which becomes clear when compared with the flat exciton line. While the detuning in this sample matches that of Sample 1 (with the PL shown Supplementary Figure 3c) the level attraction is much smaller in this sample. This result is experimental evidence that increasing the coherent coupling strength leads to reduced level attraction and thus, effects of dissipative coupling on the polariton dispersion as predicted by our model (see also Supplementary Figure 4).

Note that similar signatures of weak level attraction of excitons (or trions) and photons resulting in negative-mass dispersions were observed on $MoSe_2$ by other groups [26, 27] but their interpretation is not based on dissipative coupling. As correctly predicted by our model (see Supplementary Figure 1c and Supplementary Figure 2f of the main text), this attraction is revealed at large positive detunings $\Delta_0 > 2\sqrt{g\gamma_x}$. These multiple observations of the weak level attraction (including from Sample 4) from several groups, e.g., [26, 27], along with the theoretical calculations presented in this work, strongly suggest that dissipative exciton-photon coupling is ubiquitous in monolayer TMDCs but is often screened by the strong coherent exciton-photon coupling.

## SUPPLEMENTARY REFERENCES

* matthias.wurdack@anu.edu.au
† elena.ostrovskaya@anu.edu.au
‡ eliezer.estrecho@anu.edu.au

[1] A. Kormanyos, G. Burkard, M. Gmitra, J. Fabian, V. Zolyomi, N. D. Drummond, and V. Falko, k p theory for two-dimensional transition metal dichalcogenide semiconductors, 2D Materials **2**, 022001 (2015).

[2] X. Li, J. T. Mullen, Z. Jin, K. M. Borysenko, M. Buongiorno Nardelli, and K. W. Kim, Intrinsic electrical transport properties of monolayer silicene and $MoS_2$ from first principles, Phys. Rev. B **87**, 115418 (2013).

[3] Z. Jin, X. Li, J. T. Mullen, and K. W. Kim, Intrinsic transport properties of electrons and holes in monolayer transition-metal dichalcogenides, Phys. Rev. B **90**, 045422 (2014).

[4] M. Selig, D. Christiansen, M. Katzer, M. V. Ballottin, P. C. M. Christianen, and A. Knorr, Impact of optically pumped non-equilibrium steady states on luminescence emission of atomically-thin semiconductor excitons, arXiv:2201.03362 (2022).

[5] D. Xiao, G.-B. Liu, W. Feng, X. Xu, and W. Yao, Coupled spin and valley physics in monolayers of $mos_2$ and other group-vi dichalcogenides, Phys. Rev. Lett. **108**, 196802 (2012).

[6] M. Selig, F. Katsch, R. Schmidt, S. M. de Vasconcellos, R. Bratschitsch, E. Malic, and A. Knorr, Ultrafast dynamics in monolayer transition metal dichalcogenides: Interplay of dark excitons, phonons, and intervalley exchange, Physical Review Research **1**, 022007 (2019).

[7] A. Thränhardt, S. Kuckenburg, A. Knorr, T. Meier, and S. W. Koch, Quantum theory of phonon-assisted exciton formation and luminescence in semiconductor quantum wells, Phys. Rev. B **62**, 2706 (2000).

[8] M. Wurdack, E. Estrecho, S. Todd, T. Yun, M. Pieczarka, S. K. Earl, J. A. Davis, C. Schneider, A. G. Truscott, and E. A. Ostrovskaya, Motional narrowing, ballistic transport, and trapping of room-temperature exciton polaritons in an atomically-thin semiconductor, Nature Communications **12**, 5366 (2021).

[9] T. Yun, E. Estrecho, A. G. Truscott, E. A. Ostrovskaya, and M. J. Wurdack, Fabrication of high-quality pmma/sio x spaced planar microcavities for strong coupling of light with monolayer ws2 excitons, Applied Physics Letters **121**, 081105 (2022).

[10] K. S. Novoselov, A. K. Geim, S. V. Morozov, D. Jiang, Y. Zhang, S. V. Dubonos, I. V. Grigorieva, and A. A. Firsov, Electric field effect in atomically thin carbon films, Science **306**, 666 (2004).

[11] http://www.hqgraphene.com.

[12] http://www.novawafers.com.

[13] N. Lundt, A. Maryński, E. Cherotchenko, A. Pant, X. Fan, S. Tongay, G. Sek, A. V. Kavokin, S. Höfling, and C. Schneider, Monolayered $MoSe_2$: a candidate for room temperature polaritonics, 2D Materials **4**, 015006 (2016).

[14] Y. Yu, Y. Yu, C. Xu, Y.-Q. Cai, L. Su, Y. Zhang, Y.-W. Zhang, K. Gundogdu, and L. Cao, Engineering substrate interactions for high luminescence efficiency of transition-metal dichalcogenide monolayers, Advanced Functional Materials **26**, 4733 (2016).

[15] S. Lippert, L. M. Schneider, D. Renaud, K. N. Kang, O. Ajayi, J. Kuhnert, M.-U. Halbich, O. M. Abdulmunem, X. Lin, K. Hassoon, S. Edalati-Boostan, Y. D. Kim, W. Heimbrodt, E.-H. Yang, J. C. Hone, and A. Rahimi-Iman, Influence of the substrate material on the optical properties of tungsten diselenide

monolayers, 2D Materials **4**, 025045 (2017).

[16] A. Raja, L. Waldecker, J. Zipfel, Y. Cho, S. Brem, J. D. Ziegler, M. Kulig, T. Taniguchi, K. Watanabe, E. Malic, T. F. Heinz, T. C. Berkelbach, and A. Chernikov, Dielectric disorder in two-dimensional materials, Nature Nanotechnology **14**, 832 (2019).

[17] Z. Khatibi, M. Feierabend, M. Selig, S. Brem, C. Linderälv, P. Erhart, and E. Malic, Impact of strain on the excitonic linewidth in transition metal dichalcogenides, 2D Materials **6**, 015015 (2018).

[18] K. He, C. Poole, K. F. Mak, and J. Shan, Experimental demonstration of continuous electronic structure tuning via strain in atomically thin $MoS_2$, Nano Letters **13**, 2931 (2013).

[19] J. Martín-Sánchez, A. Mariscal, M. D. Luca, A. T. Martín-Luengo, G. Gramse, A. Halilovic, R. Serna, A. Bonanni, I. Zardo, R. Trotta, and A. Rastelli, Effects of dielectric stoichiometry on the photoluminescence properties of encapsulated $WSe_2$ monolayers, Nano Research **11**, 1399 (2018).

[20] T. Yun, M. Wurdack, M. Pieczarka, S. Bhattacharyya, Q. Ou, C. Notthoff, C. K. Nguyen, T. Daeneke, P. Kluth, M. S. Fuhrer, A. G. Truscott, E. Estrecho, and E. A. Ostrovskaya, Influence of direct deposition of dielectric materials on the optical response of monolayer $WS_2$, Applied Physics Letters **119**, 133106 (2021).

[21] T. P. Lyons, D. J. Gillard, C. Leblanc, J. Puebla, D. D. Solnyshkov, L. Klompmaker, I. A. Akimov, C. Louca, P. Muduli, A. Genco, M. Bayer, Y. Otani, G. Malpuech, and A. I. Tartakovskii, Giant effective Zeeman splitting in a monolayer semiconductor realized by spin-selective strong light–matter coupling, Nature Photonics **16**, 632 (2022).

[22] J. Jadczak, J. Kutrowska-Girzycka, P. Kapuściński, Y. S. Huang, A. Wójs, and L. Bryja, Probing of free and localized excitons and trions in atomically thin WSe2, WS2, MoSe2 and MoS2 in photoluminescence and reflectivity experiments, Nanotechnology **28** (2017).

[23] C. Rupprecht, N. Lundt, M. Wurdack, P. Stepanov, E. Estrecho, M. Richard, E. A. Ostrovskaya, S. Höfling, and C. Schneider, Micro-mechanical assembly and characterization of high-quality Fabry–Pérot microcavities for the integration of two-dimensional materials, Applied Physics Letters **118**, 103103 (2021).

[24] The fabrication of the high-reflectivity substrate was performed at the ACT OptoFab node of the Australian National Fabrication Facility - a company established under the National Collaborative Research Infrastructure Strategy to provide nano and microfabrication facilities for Australia's researchers.

[25] M. Wurdack, E. Estrecho, S. Todd, C. Schneider, A. G. Truscott, and E. A. Ostrovskaya, Enhancing Ground-State Population and Macroscopic Coherence of Room-Temperature $WS_2$ Polaritons through Engineered Confinement, Phys. Rev. Lett. **129**, 147402 (2022).

[26] S. Dhara, C. Chakraborty, K. M. Goodfellow, L. Qiu, T. A. O'Loughlin, G. W. Wicks, S. Bhattacharjee, and A. N. Vamivakas, Anomalous dispersion of microcavity trion-polaritons, Nature Physics **14**, 130 (2018).

[27] E. Y. Paik, L. Zhang, S. Hou, H. Zhao, Y.-H. Chou, S. R. Forrest, and H. Deng, High quality factor microcavity for van der waals semiconductor polaritons using a transferrable mirror, Advanced Optical Materials **11**, 2201440.
